# Periodontal pathogens and tetracycline resistance genes in subgingival biofilm of periodontally healthy and diseased Dominican adults

**DOI:** 10.1007/s00784-015-1516-2

**Published:** 2015-06-30

**Authors:** James R. Collins, Alexandre Arredondo, Alma Roa, Yleana Valdez, Rubén León, Vanessa Blanc

**Affiliations:** Department of Periodontics, School of Dentistry, Pontificia Universidad Católica Madre y Maestra (PUCMM), Santo Domingo, Dominican Republic; Department of Microbiology, Dentaid Research Center, Dentaid S. L., Ronda Can Fatjò 10, Parc Tecnològic del Vallès, 08290 Barcelona, Spain; Department of Periodontology, Graduate School, Universidad Católica Santo Domingo, Santo Domingo, Dominican Republic

**Keywords:** Periodontopathogens, Antimicrobial resistance genes, Dominican Republic

## Abstract

**Objective:**

The objective of this study was to compare the periodontopathogen prevalence and tetracycline resistance genes in Dominican patients with different periodontal conditions.

**Methods:**

Seventy-seven samples were collected from healthy, gingivitis, chronic (CP) and aggressive (AgP) periodontitis patients. *Porphyromonas gingivalis*, *Treponema denticola*, *Tannerella forsythia*, *Aggregatibacter actinomycetemcomitans*, *Fusobacterium nucleatum*, *Prevotella intermedia*, *Parvimonas micra*, *Eikenella corrodens* and *Dialister pneumosintes* and 11 resistance genes were studied by PCR. *P. gingivalis* fimA genotype was determined.

**Results:**

In healthy patients, *P. micra* and *P. intermedia* were the most and least frequently detected, respectively. *T. forsythia* and *E. corrodens* appeared in 100 % of gingivitis patients. Red complex, *D. pneumosintes* and *E. corrodens* were significantly more prevalent in CP compared to healthy patients. *F. nucleatum* and *T. denticola* were detected more frequently in AgP. *A. actinomycetemcomitans* was the most rarely observed in all groups. The fimA II genotype was the most prevalent in periodontitis patients. Seven tetracycline-resistant genes were detected. *tet*(*Q*), *tet*(*32*) and *tet*(*W*) showed the greatest prevalence. *tet*(*32*) was significantly more prevalent in CP than in healthy patients.

**Conclusions:**

Red complex bacteria and *D. pneumosintes* were significantly the most prevalent species among periodontitis patients. *T. forsythia* was the most frequently detected in this population. To our knowledge, this is the first study describing the *tet*(*32*) gene in subgingival biofilm from healthy and periodontally diseased subjects.

**Clinical relevance:**

This study contributes to the knowledge on the subgingival microbiota and its resistance genes of a scarcely studied world region. Knowing the prevalence of resistance genes could impact on their clinical prescription and could raise awareness to the appropriate use of antibiotics.

**Electronic supplementary material:**

The online version of this article (doi:10.1007/s00784-015-1516-2) contains supplementary material, which is available to authorized users.

## Introduction

Periodontal disease is the result of an imbalance in the microbial ecology of the oral cavity, and it also depends on host susceptibility [1]. The prevalence of this disease is very high in most populations, and gingivitis may affect up to 100 % of the population [2]. Factors such as socio-economic background, gender, smoking, lifestyle or stress appear to have an impact on the prevalence and severity of periodontitis in any given population [3].

Clinical-microbiological studies have shown a close association between periodontitis and the presence of a specific group of microorganisms, mainly *Porphyromonas gingivalis*, *Aggregatibacter actinomycetemcomitans*, *Treponema denticola* and *Tannerella forsythia* [1, 2]. The prevalence of these periodontal pathogens has been established in developed countries, and only in recent years has prevalence data of this type been determined in some developing countries [2]. Comparisons of the periodontitis-associated microbiota in different world regions suggest that the distribution of periodontopathogens depends upon geographic location and patient ethnic group [2, 4]. For example, significant differences have been reported for the prevalence of *A. actinomycetemcomitans* in different European populations [5]. Subjects of different ethnic origins living in the USA have also shown different prevalences of this microorganism [6]. This species has also been described to show a high frequency in populations of China and North Africa [7, 8] and a low prevalence in Japan [9], USA [10] and Chile [11]. Moreover, inter- and intra-population clonal variability for different virulence factors has been observed in this microorganism, [2, 12, 13].

Differences in prevalence have also been described for *P. gingivalis* in different ethnic groups [2]. And similarly for this species, genetic variability has been reported in genes related with the synthesis of some virulence factors, for example, the locus responsible for the synthesis of the capsule [14] and the gene responsible for the synthesis of the fimbria (*fimA*). Six genotypes have been described for the *fimA* gene, and genotypes II and IV have been associated with periodontitis [2, 15, 16].

Moreover, an increase in multi-resistant strains in recent years has led to considerable concern throughout the different medical disciplines and has augmented the need to learn about the mechanisms and genes involved in the said process [17, 18]. Relatively little information exists relating to resistance patterns or to the resistance genes that could be present in subgingival microbiota. Recent studies have reported periodontal pathogens resistant to different antibiotics, with percentages that vary according to the populations studied; this could be due to the lack of control over antibiotic use and to poor patient treatment compliance in certain countries [5, 19, 20]. Tetracycline and some of its derivatives have been widely used as adjuvants in the treatment of periodontitis, both topically and systemically [21, 22]. Studies conducted in Europe and the USA report a large presence of resistance genes to this antibiotic in microorganisms from the oral cavity, some of whose frequencies differ, depending on the population studied.

Currently, there is only one study in the Dominican Republic on periodontal microbiota, involving 24 patients [23]. Using culture techniques, a predominance of enteric bacteria and low prevalence and diversity of periodontopathogens were described. Furthermore, in the Dominican Republic, there have been no studies describing the presence of antibiotic resistance genes in subgingival microbiota. In view of the above, the purpose of this study was to describe (i) the prevalence of periodontopathogens, (ii) the *fimA* gene type present in *P. gingivalis*-positive samples and (iii) the frequency of tetracycline resistance genes in subgingival samples of healthy Dominican patients and those affected by gingivitis, chronic periodontitis (CP) and aggressive periodontitis (AgP).

## Material and methods

### Subject population

Patients were selected from the Graduate School of Dentistry at the Catholic University of Santo Domingo, Dominican Republic, between 2011 and 2012. Their clinical conditions were defined according to the American Academy of Periodontology’s Periodontal Disease Classification Consensus Report [24]. A total of 77 subjects were recruited (Table 1). All subjects signed an institutional review board (IRB)-approved informed consent form. The protocol and study design were previously approved by the university’s IRB. Patients with a diagnosis of gingivitis, CP and AgP were considered candidates with each individual having at least 12 teeth, not counting third molars. Exclusion criteria included cancer, cardiovascular disease, HIV infection, diabetes, arthritis, pulmonary disease or any other systemic disease. Pregnant women, subjects who had taken antibiotics and/or anti-inflammatory drugs within 6 months prior to the study, heavy smokers (>10 cigarettes per day) and patients with a previous periodontal treatment were also excluded.

**Table 1:** Clinical description of study sample, mean ± standard deviation

	Healthy *N* = 11	Gingivitis *N* = 10	CP *N* = 30	AgP *N* = 26
Age (range)	28.3 ± 7.9 (24–52)	36.30 ± 11.50 (20–56)	45.7 ± 10.1 (27–65)	33.9 ± 7.7 (18–46)
Females (*N*)	9	8	17	19
Sites with plaque (%)	2.2 ± 0.9	30 ± 4	54 ± 19	39 ± 17
Probing depth (mm)	1.5 ± 0.3	2.5 ± 0.5	4.4 ± 1.3	5.9 ± 2.2
Probing depth at samples site (mm)	2.1 ± 0.4	2.9 ± 0.2	5.6 ± 1.1	8.2 ± 1.5
CAL (mm)	0.4 ± 0.2	2.5 ± 0.4	5.1 ± 1.5	6.5 ± 2.1

### Clinical procedures

A complete periodontal examination, excluding third molars, was performed on all subjects. The following clinical parameters were recorded: plaque index (PI), percentage of sites with bleeding on probing (BOP), probing depth (PD) in millimetres and clinical attachment level (CAL) in millimetres. Six sites were examined for each tooth: mesio-buccal, buccal, disto-buccal, disto-lingual, lingual and mesio-lingual using a North Carolina periodontal probe. Periodontal patients were described as having a minimum of four sites with a pocket depth ≥4 mm and radiographic evidence of alveolar bone loss.

### Subgingival bacterial sampling and processing

Subgingival samples from CP and AgP were taken from the four deepest periodontally affected sites with a probing depth of ≥4 mm, one in each quadrant [25]. Sampled sites in healthy and gingivitis patients had a probing depth of ≤3 mm, with the deepest sites in each quadrant being selected. After isolating the area with cotton rolls, supragingival deposits were carefully removed with curettes and subgingival microbial samples were obtained by inserting sterile paper points to the bottom of the periodontal pocket and keeping them in place for 20 s. Paper points were pooled in a sterile vial, and samples were sent to the microbiology laboratory (Dentaid Research Center, Barcelona, Spain) at a controlled temperature of −20 °C for processing. Genomic DNA was isolated from subgingival samples using a QIAamp kit (Qiagen, Hilden, Germany) following the manufacturer’s instructions. Samples were previously treated with lysozyme (20 mg/mL) for 7 min at 37 °C. The DNA concentration was quantified using a Nanodrop® ND-1000 UV–Vis spectrophotometer (Nanodrop Technologies, Wilmington, DE, USA).

### Bacterial detection by PCR and *fimA* gene type identification

16S rDNA from *P. gingivalis*, *T. denticola*, *T. forsythia*, *A. actinomycetemcomitans*, *P. intermedia* and *Eikenella corrodens* was amplified using specific primers described by Ashimoto et al. [10]. Specific primers for *Parvimonas micra* (Pm1: 5′-CATgCAAgTCgAACgTgATT-3′ and Pm2: 5′-CgAAAACCTTCTTCgCTCAC-3′) and *Fusobacterium nucleatum* (Fn1: 5′-TAAAgCgCgTCTAggTggTT-3′ and Fn2: 5′-ACggCTTTgCAACTCTCTgT-3′) were designed using the NCBI Reference Sequences NR_114338.1 and NR_074412.1, respectively. Possible specific regions for primer design were found through multiple alignments with other oral bacterial sequences (ClustalW2 programme, http://www.ebi.ac.uk). Primers were designed using the primer-Blast programme (http://www.ncbi.nlm.nih.gov/tools/primer-blast/). Specificity was tested by PCR, using total DNA extracted from 15 collection strains as template, where only *P. micra* DSM 20468 and *F. nucleatum* DSM 20482 DNA tested positive. The limit of detection was 10^2^ cfu/ml which was determined using the dilution of pure cultures from both strains.

*Dialister pneumosintes* primers and PCR conditions were described by Doan et al. [26]. PCR mixes were performed in 25 μL containing 100 ng of template, 1x PCR buffer, 2.5 mM of MgCl_2_, 0.2 mM of each dNTP, 1 μM of each primer and 0.5 U Taq Expand DNA polymerase.

The PCR amplification programme was standardised and included initial DNA denaturation at 95 °C for 5 min and a total of 35 PCR cycles and each cycle consisting of 1 min of denaturation at 95 °C, 1 min of annealing at 55 °C and 1 min of extension at 72 °C. Finally, an extension step was applied at 72 °C for 10 min. Sterilised Milli-Q water was used as template for negative control in each reaction set, and pure DNA from the following strains was used as positive control reactions: *P. gingivalis* ATCC 33277, *A. actinomycetemcomitans* DSM 8324, *F. nucleatum* DSM 20482, *P. intermedia* NCTC 13070, *T. forsythia* ATCC 43037, *P. micra* DSM 20468, *T. denticola* ATCC 35405, *E. corrodens* NCTC 10596 and *D. pneumosintes* ATCC 33048. PCR reactions were done in duplicate. Amplification results were evaluated by electrophoresis using 4 % agarose gel with ethidium bromide (0.5 g/mL) in 1x TAE buffer. The 100 bp DNA Ladder (New England Biolabs, Inc. Ipswich, MA, USA) served as the molecular weight marker. The gels were photographed using a UV light transilluminator GEL DOC™ XR+ system (Bio-Rad Laboratories Inc. Hercules, CA, USA).

*fimA* gene type (I, Ib, II, III, IV and V) identification was carried out using specific primers and PCR conditions described by Amano et al. [16] and Nakagawa et al. [27]. The negative control was sterile Milli-Q water.

### Detection of tetracycline resistance gene

The tetracycline resistance genes were detected performing multiplex PCR with 26 primers (Table 2). PCR mixes were performed in 25 μL containing 100 ng of template, 1x PCR buffer, 1.0 mM of each dNTP, 0.5 μM of each primer and 1.0 U Taq Expand DNA polymerase. The MgCl_2_ was 2.0 mM for mixes 1 and 2, and 2.5 mM for mixes 3 and 4. The amplification programmes were as follows: 30 PCR cycles of (i) mix 1: 45 s of denaturation at 95 °C, 45 s of annealing at 64 °C and 70 s of extension at 72 °C; (ii) mix 2: 45 s of denaturation at 95 °C, 45 s of annealing at 60 °C and 90 s of extension at 72 °C; (iii) mix 3: 30 s of denaturation at 95 °C, 30 s of annealing at 58 °C and 60 s of extension at 72 °C; and (iv) mix 4: 30 s of denaturation at 95 °C, 30 s of annealing at 56.5 °C and 45 s of extension at 72 °C.

**Table 2:** List of primers for detection of different resistance genes. It also indicates the multiplex group and expected amplicon sizes

Multiplex group	Primer	Sequence (5′-3′)	Product size (bp)	Source
1	TetM F TetM R	GCG TAC AAG CAC AGA CTC GT AGC CAT AGC GTA TCC CCT CC	1142	This study
	TetW F TetW R	GAG AGC CTG CTA TAT GCC AGC GGG CGT ATC CAC AAT GTT AAC	168	[28]
2	TetQ F TetQ R	AGA ATC TGC TGT TTG CCA GTG CGG AGT GTC AAT GAT ATT GCA	167	[28]
	Tet32 F Tet32 R	GAA CCA GAT GCT GCT CTT CAT AGC CAC GCC CAC ATG AT	620	[29]
	TetL F TetL R	TCG TTA GCG TGC TGT CAT TC GTA TCC CAC CAA TGT AGC CG	267	[30]
3	TetS F TetS R	GAA AGC TTA CTA TAC AGT AGC AGG AGT ATC TAC AAT ATT TAC	168	[28]
	TetO F TetO R	AAC TTA GGC ATT CTG GCT CAC TCC CAC TGT TCC ATA TCG TCA	515	[31]
	Tet31 F Tet31 R	CAA TCA CGC CCA AAA GAA TGT GCC ATC CCA GTT TGT	564	[32]
4	TetB F TetB R	AAT AGC CAC TAA ATG GGG CG ATA ACA CCG GTT GCA TTG GT	243	This study
	TetK F TetK R	TCG ATA GGA ACA GCA GTA CAG CAG ATC CTA CTC CTT	169	[30]
	Tet37 F Tet37 R	ATG GTT CGC TAT TAC TCT AAC ATC AGT CTC ATA TTT CGA CA	177	This study

Negative and positive controls were used for each PCR reaction. All reagents used for PCR reactions were purchased from Roche Diagnostics (Penzberg, Germany). Amplification reactions were carried out in a Thermal cycler T3000 (Biometra, Goettingen, Germany).

### Statistical analysis

The statistical analysis was performed using SAS System® v9.2 (SAS Institute Inc., Cary, NC, USA). For statistical tests, a nominal significance level of 5 % (*P* < 0.05) was applied. Standard methods were used to calculate descriptive statistics. The association between bacterial detection and clinical parameters was tested using the Mann-Whitney for continuous variables and chi-square or Fisher’s exact tests for categorical factors. In order to compare the prevalence of each species, a logistic regression method was performed for each diagnostic group. Multiple comparisons with Tukey adjustment were also performed. To assess whether a population’s prevalence of some bacteria was different from the observed value of 100 %, the binomial test and Clopper-Pearson confidence limits were calculated. Similarly, the chi-square test and a logistic regression method were performed to assess differences in prevalence of *tet* genes between the four diagnostic groups.

## Results

Table 1 describes the clinical and demographic characteristics of the study subjects. No statistically significant differences in gender existed between groups. However, significant differences were observed between healthy and periodontitis patients in relation to probing depth (*p* < 0.001) and CAL (*p* < 0.001) and to the percentage of sites with plaque (*p* < 0.0001). Patients with AgP showed greater PD (*p* < 0.0048) and CAL (*p* < 0.04) than patients with CP.

Averages of the total bacteria detected for each patient group were as follows: healthy, 2.8 ± 1.6; gingivitis, 5.9 ± 1.3; CP, 6.9 ± 1.8; and AgP, 6.7 ± 1.7. In addition, the use of the Mann-Whitney-Wilcoxon *U* test determined that the bacterial average in the healthy subjects was significantly lower than in the other groups, gingivitis (*p* = 0.0189), CP (*p* = 0.0005) and AgP (*p* = 0.0011). Red complex bacteria and *F. nucleatum*, *P. micra* and *E. corrodens* were detected in more than 70 % of the patients studied (in all diagnostic groups). However, the distribution of these species was not the same throughout the four groups (Pearson’s chi-square, *p* < 0.05) (Table 3).

**Table 3:** Number and percentage of positive subjects for each microorganism in the four studied groups

Bacteria	Healthy (*n* = 11)		Gingivitis (*n* = 10)		CP (*n* = 30)		AgP (*n* = 26)		Total (*n* = 77)	
	*N* ^a^	%^b^	*N* ^a^	%^b^	*N* ^a^	%^b^	*N* ^a^	%^b^	*N* ^a^	%^b^
*P. gingivalis*	2	18.2	7	70	28	93.3^*^	23	88.5^*^	60	77.9
*T. denticola*	3	27.3	9	90	27	90^*^	24	92.3^*^	63	81.8
*T. forsythia*	6	54.5	10	100	29	96.7^*^	24	92.3	69	89.6
*A. actinomycetemcomitans*	2	18.2	1	10	10	33.3	6	23.1	19	24.7
*P. intermedia*	0	0	2	20	16	53.3	7	26.9	25	32.5
*F. nucleatum*	5	45.4	9	90	24	80	24	92.3^*^	62	80.5
*P. micra*	7	63.6	8	80	27	90	23	88.5	65	84.4
*D. pneumosintes*	1	9.1	3	30	20	66.7^*^	22	84.6^*^	46	59.7
*E. corrodens*	5	45.4	10	100	27	90^*^	21	80.8	63	81.8
Mean bacteria in each group of patients (±SD)	2.8 ± 1.6		5.9 ± 1.3		6.9 ± 1.8		6.7 ± 1.7			

^a^ *N* number of positive subjects for each bacterium

^b^ *%* percentage of positive subjects for each bacterium

*P values <0.05 obtained from statistical comparison between the prevalence data of healthy and diseased subjects

In healthy subjects, the most frequent bacterium was *P. micra* (63.6 %), although it is worth mentioning that *T. forsythia* was found in over 50 % of the healthy population. Furthermore, *P. intermedia* was not found in this group.

*P. gingivalis*, *T. denticola* and *T. forsythia* were the most prevalent in periodontally diseased patients. *P. gingivalis* and T*. denticola* were significantly more prevalent in diseased patients compared to healthy patients. The detection frequency of *T. denticola* in the healthy patient group was significantly lower than in the CP and AgP patient groups, with *p* values of 0.0045 and 0.0048, respectively. And the same is observed when studying the percentages of *D. pneumosintes*, as this species was found mainly in patients with aggressive (84.6 %) and chronic periodontitis (66.7 %). *T. forsythia* was frequently detected in all groups studied, although it was significantly more prevalent in patients with chronic periodontitis than in healthy patients. Moreover, the Clopper-Pearson test showed statistical differences between the gingivitis group and the healthy groups for this species. *E. corrodens* behaved similarly to *T. forsythia* (Table 3).

*F. nucleatum*, *T. forsythia* and *T. denticola* had over 92 % occurrence in AgP and were the most prevalent species in this group. Of these three species, *F. nucleatum* and *T. denticola* were much more frequently detected in these patients than in healthy subjects. However, *A. actinomycetemcomitans* was the least prevalent species in all patient groups, and no significant differences were observed for this species between the healthy and diseased subjects. This bacterium was only present in 24.7 % of the entire test population (Table 3).

Of the total patients included in the study, 77.9 % (*n* = 60) tested positive for *P. gingivalis*. In 57 of these, the *fimA* gene variant was determined. The III, V and Ib genotypes were not detected in any of the patients. By using Pearson’s chi-squared test, it was determined that *fimA* I, II and IV genotypes were not equally distributed across the four groups (*p* = 0.0003). The genotype II frequency was significantly higher in AgP (84 %) and CP (75.8 %) patients.

The presence of 11 tetracycline resistance genes was analysed. *tet*(*S*), *tet*(*31*), *tet*(*37*) and *tet*(*K*) genes were not detected. *tet*(*L*) and *tet*(*B*) were only detected in one CP patient. This set of six genes was excluded from subsequent statistical analysis. The weighted average number of genes per patient showed that in healthy subjects the value (1.47) was one point lower than that obtained in patients with chronic periodontitis (2.88) (Table 4). Lastly, we observed that the most frequently detected gene in this population was *tet*(*Q*), which was present in 54 of the 75 patients tested. And when comparing resistance gene prevalence in the different patient groups, significant differences were found for the *tet*(*Q*) and *tet*(*32*) genes. We observed that these genes were much more prevalent in CP than in the healthy patient group (*p* = 0.023 and *p* = 0.019, respectively).

**Table 4:** Prevalence of antibiotic resistance genes in subgingival samples from the different periodontal conditions

Gene	Healthy		Gingivitis		CP		AgP	
	P/A	%	P/A	%	P/A	%	P/A	%
*tet*(*31*)	0/11	–	0/9	–	0/29	–	0/26	–
*tet*(*32*)	1/11	9.09	6/9	66.7	21/29	72.4	16/26	61.5
*tet*(*37*)	0/11	–	0/9	–	0/29	–	0/26	–
*tet*(*B*)	0/11	–	0/9	–	1/17	5.88	0/26	–
*tet*(*K*)	0/11	–	0/9	–	0/29	–	0/26	–
*tet*(*L*)	0/11	–	0/9	–	1/29	3.45	0/26	–
*tet*(*M*)	5/11	45.5	1/9	11.1	9/29	31.0	7/26	26.9
*tet*(*O*)	1/9	11.1	2/6	33.3	9/29	31.0	2/23	8.70
*tet*(*Q*)	5/11	45.5	6/9	66.7	24/29	82.8	19/26	73.1
*tet*(*S*)	0/11	–	0/9	–	0/29	–	0/26	–
*tet*(*W*)	4/11	36.4	2/9	22.2	18/29	62.1	17/26	65.4
WAN genes/patient	1/48		2.0		2.89		2.36	

Due to a lack of samples, not all the patients could not be analysed

*WAN* weighted average number of genes per patient, *P/A* positive/all

## Discussion

In this cross-sectional study, we used PCR to determine the prevalence of nine periodontopathogens in the subgingival microbiota of 77 Dominican patients diagnosed as healthy, gingivitis, CP and AgP. The *fimA* genotypes were determined in *P. gingivalis-*positive patients. Finally, the frequency of 11 tetracycline resistance genes was also analysed.

Little information is available regarding subgingival microbiota in Central America and the Caribbean countries. In the Dominican Republic, only one work exists, in which by using culturing methods, the subgingival microbiota of 24 patients with advanced periodontitis were studied. In the said study, a high rate of enteric bacteria was found and the only member of the red complex detected was *P. gingivalis.* A low prevalence of spirochetes was also observed [23]. Our study, using PCR, showed an incidence of red complex microorganisms of around 90 % in periodontitis patients. The high prevalence of *P. gingivalis* in the CP group was similar to that obtained in other studies performed in Colombia [33], Chile [11] and Spain [34]. Although in our case, no significant differences were found between the CP and AgP patient groups.

In subjects with CP, *T. forsythia* was the most frequently detected species (96.7 %), which differs from that which is described in other Latin American countries (Chile [16.2 %] and Colombia [39 %]) [34] and in European countries, including the Netherlands (73.3 %) [5], UK (44.4 %) [35] and Spain (36.1 %) [34]. A high incidence of *T. forsythia* was also observed in the healthy (54.5 %) and gingivitis (100 %) groups. In Thai urban adults, Torrungruang et al. [36] observed a high prevalence of *T. forsythia* in healthy and CP patients, and they were not able to associate this microorganism with this disease. Further studies may be necessary to establish clear associations between *T. forsythia* and periodontitis in the Dominican population. It is likely that the use of PCR in our study allowed us to observe this species more frequently compared to other studies that only employed culture techniques [37, 38].

*D. pneumosintes* is a species that is frequently found in deep sites and in severe forms of the disease [26, 39], a fact that coincides with our study, as it was detected more frequently in patients with periodontitis compared to healthy or gingivitis patients. Even so, the high colonisation of this species in the Dominican Republic contrasts with that described by Botero et al. in Colombia [33] and is similar to that found in Brazil [39]. That being said, some studies report a frequency for this species of greater than 70 % in healthy patients [40].

The literature indicates that there are differences between the subgingival microbiota of the CP and AgP groups [24], and further emphasises the strong association between the presence of *A. actinomycetemcomitans* and AgP [41, 42]. In our study, no significant differences were observed between the species studied in the CP and AgP groups. Even *A. actinomycetemcomitans* was less frequently detected in AgP than in CP. The frequency of this species in the Dominican Republic was similar to that reported in Chile (19.4 %) [11] and higher than that described in Colombia (8.3 %) [33]. High prevalence of this bacterium has been reported for AgP in other populations: 60.4 % in Morocco [8], 62 % in China [7], 75 % in Korea [43], 80 % in Brazil [44] and 84.3 % in Taiwan [45]. This may be due to ethnic differences between populations [2, 3].

Some studies conducted in Brazil have found a high frequency of the JP2 clone of *A. actinomycetemcomitans* [46, 47]; this overproducer of leukotoxin has been closely associated in some populations with AgP [48]. In our study, we attempted to determine the presence of this clone, although the sample amount only allowed for testing four *A. actinomycetemcomitans-*positive samples (data not shown). Nevertheless, we were able to observe the genetic variability of the *fimA* gene of *P. gingivalis*. Fifty-seven of the 60 *P. gingivalis*-positive samples were studied, and *fimA* type II was the most frequently found allele in periodontitis patients (75.9 % [CP]; 84 % [AgP]). This is in agreement with other studies where genotypes II and IV are more frequently detected in periodontitis patients than in healthy ones [2, 16, 49].

The oral cavity has been described as a reservoir of potentially transferable antibiotic resistance genes [50]. In the Dominican Republic, there are no policies controlling the sale of antibiotics, and the livestock industry is authorised to use subtherapeutic levels of antibiotic as growth promoters in poultry feed [51]. This can result in the emergence of resistant or multi-resistant bacterial strains [52]. Tetracyclines have been used as a therapy for periodontitis as a monotherapy or as an adjunct to scaling and root planing with different degrees of efficacy [22]. Although nowadays it is not used regularly, some of its derivatives are used in subantimicrobial doses for the downregulation of metalloproteinase activity [53]. Studies have been carried out in Europe and the USA to analyse the subgingival presence of these resistance genes in healthy and periodontally diseased subjects [50]. In our study, we have tested for genes encoding efflux pumps: *tet*(*31*), *tet*(*K*), *tet*(*L*), ribosomal protection proteins (RPP): *tet*(*B*), *tet*(*M*), *tet*(*O*), *tet*(*Q*), *tet*(*S*), *tet*(*W*) and *tet*(*32*) and enzymatic inactivation *tet*(*37*). This covers the different resistance mechanisms described for this antibiotic. Several of these genes have been detected in bacteria from the oral cavity, while for other genes no references exist in the literature in relation to the oral environment [50, 54]. In our study, the most prevalent gene in periodontitis was *tet*(*Q*) while *tet*(*M*) showed a low prevalence. However, studies by Ioannidis et al. [55] and Kim et al. [56] reported both genes as the most frequent in diseased patients in Europe and the USA, respectively. In Dominican patients, *tet*(*32*) was the second most frequently detected gene in periodontitis; it is worth noting that, to our knowledge, this is the first study to report the presence of this gene in the subgingival biofilm of healthy and periodontally diseased patients.

The results of this study show a high prevalence of red complex organisms in patients with CP and AgP. However, the high incidence of *T. forsythia* in healthy and gingivitis patients may suggest further study is needed to determine the role of this species in the Dominican population.

Unfortunately, a larger number of patients was not possible for this study. Even so, and considering the existing knowledge on subgingival microbiota and the presence and distribution of antibiotic resistance genes in the Dominican Republic and Central America, we believe that this study provides interesting data in relation to the subgingival microbiota of this population, as some differences were found compared to other studies performed in populations in America and Europe.

Moreover, we found a wide variety of tetracycline resistance genes with prevalences differing from those described in European and North American populations. Considering the current public health concern surrounding the presence of multi-resistant bacteria caused by the use and abuse of antibiotics [18], it would be wise to conduct broader studies, allowing us to map the diversity, distribution and dissemination of resistance genes in different countries in America.

## Electronic supplementary material

ESM 1 (DOCX 6.55 mb)

ESM 2 (DOCX 3.76 mb)

ESM 3 (DOCX 4.77 mb)

ESM 4 (DOCX 725 kb)
