# Supplementary material for: Periodontal pathogens and tetracycline resistance genes in subgingival biofilm of periodontally healthy and diseased Dominican adults
Source: Clin Oral Investig. 2015 Jun 30;20:349–56. doi: 10.1007/s00784-015-1516-2 (PMC4762914; doi:10.1007/s00784-015-1516-2)
Supplement: Supplementary file 1 — (DOCX 6.55 mb) [file 784_2015_1516_MOESM1_ESM.docx]

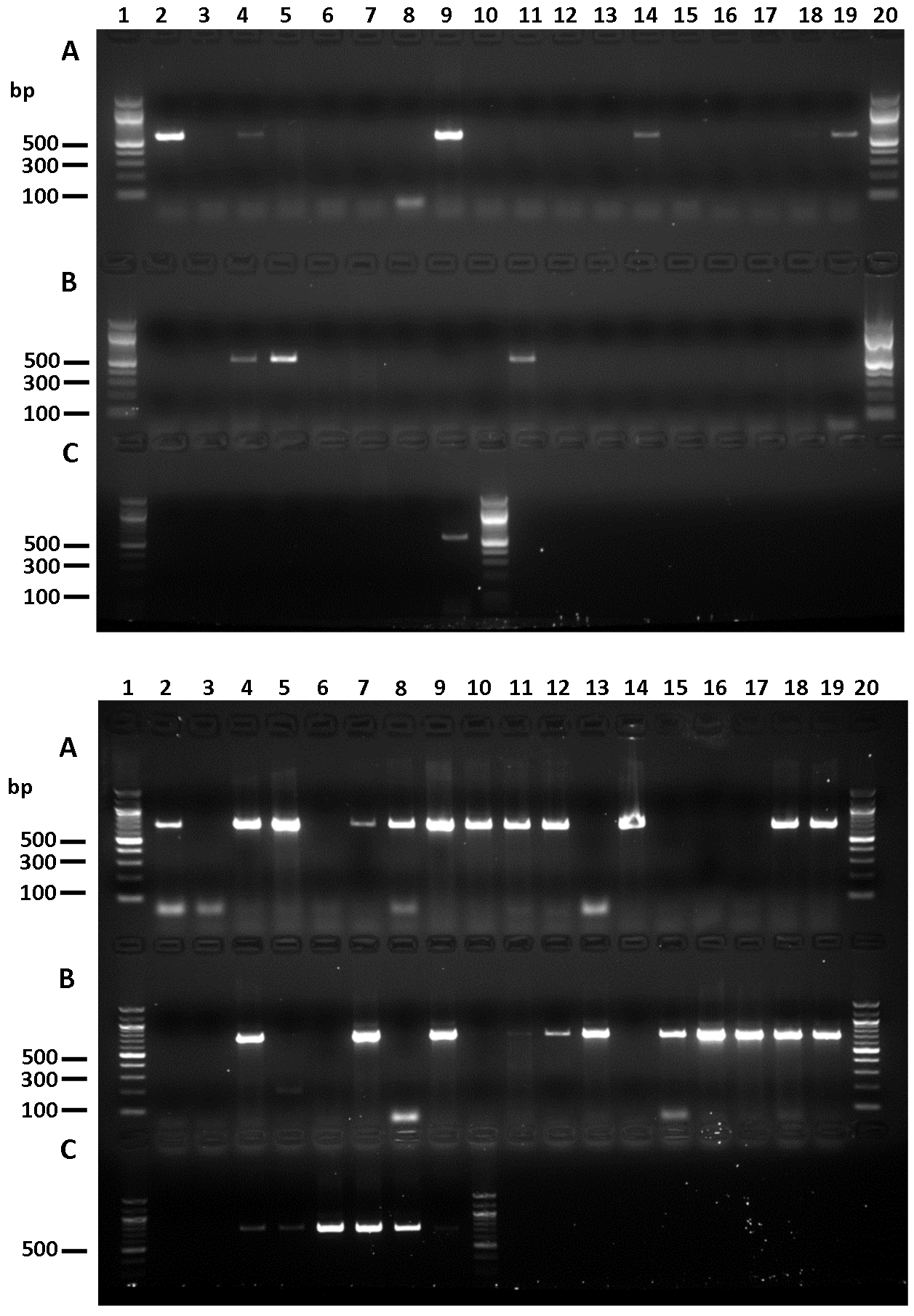


**I**

**II**

Figure 1.- PCR detection of periodontal bacteria in samples from Dominican Republic patients. In I detection of *Aggregatibacter actinomycetemcomitans*. In II detection of *Fusobacterium nucleatum*. For both gels, Lane A2 corresponds to positive controls done using DNA from the *A. actinomycetemcomitans* DSM 8324 and *F. nucleatum* DSM 20482 type strains. Likewise, Lanes A3 correspond to negative controls. Lane 1 and 20, 100-base pair DNA ladder marker.
