# Supplementary material for: Periodontal pathogens and tetracycline resistance genes in subgingival biofilm of periodontally healthy and diseased Dominican adults
Source: Clin Oral Investig. 2015 Jun 30;20:349–56. doi: 10.1007/s00784-015-1516-2 (PMC4762914; doi:10.1007/s00784-015-1516-2)
Supplement: Supplementary file 2 — (DOCX 3.76 mb) [file 784_2015_1516_MOESM2_ESM.docx]

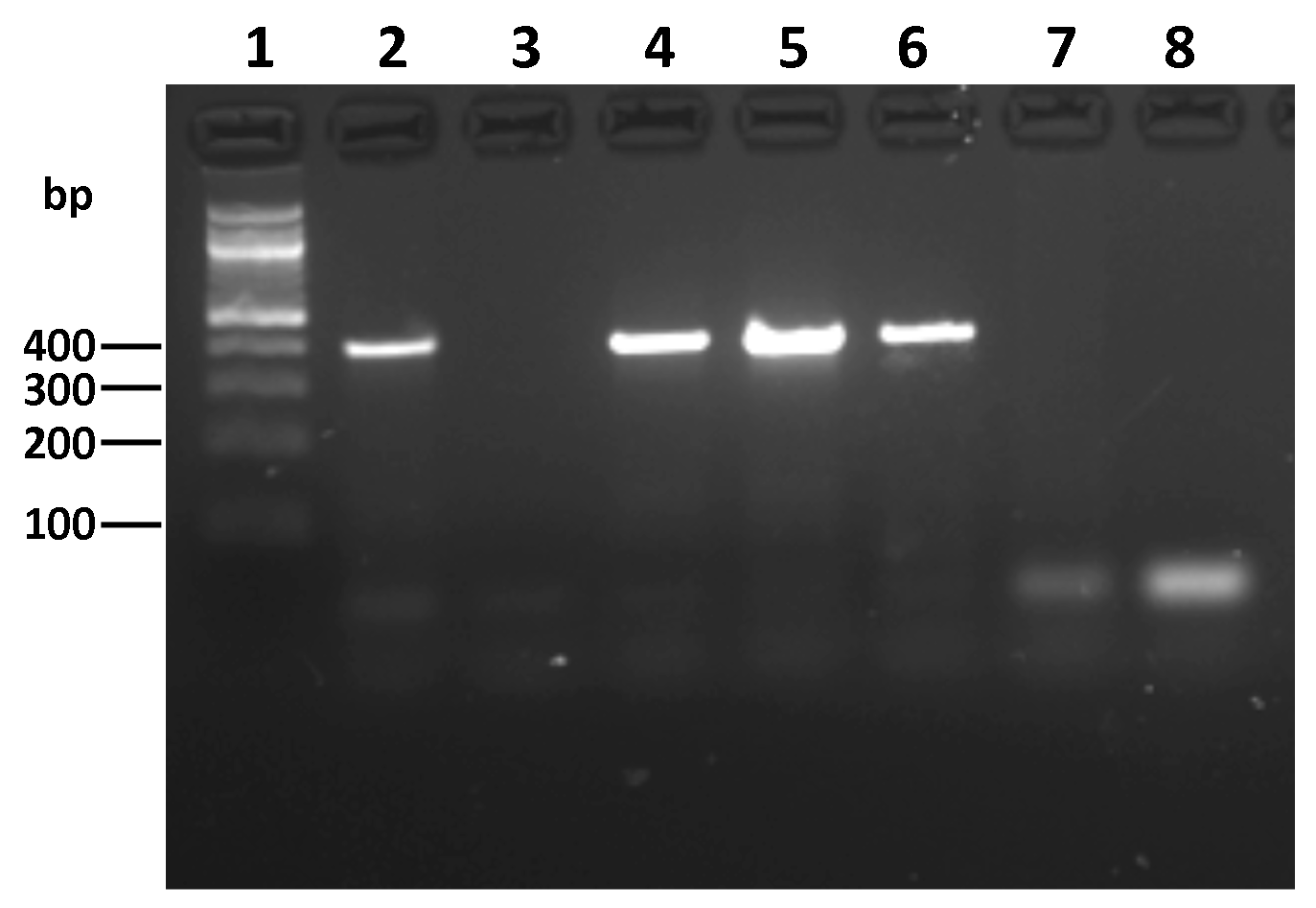


Figure 2.- fimA I genotype of *P. gingivalis* positives samples. Lanes: 2), 3), 4), 5), 6) y 7) corresponds to the 13, 16, 30, 33, 40 and 44 samples, respectively. Lane 8) Negative control. Lane 1: 100-base pair DNA ladder marker. Agarose gel electrophoresis at 4%.
