# Supplementary material for: Periodontal pathogens and tetracycline resistance genes in subgingival biofilm of periodontally healthy and diseased Dominican adults
Source: Clin Oral Investig. 2015 Jun 30;20:349–56. doi: 10.1007/s00784-015-1516-2 (PMC4762914; doi:10.1007/s00784-015-1516-2)
Supplement: Supplementary file 3 — (DOCX 4.77 mb) [file 784_2015_1516_MOESM3_ESM.docx]

**
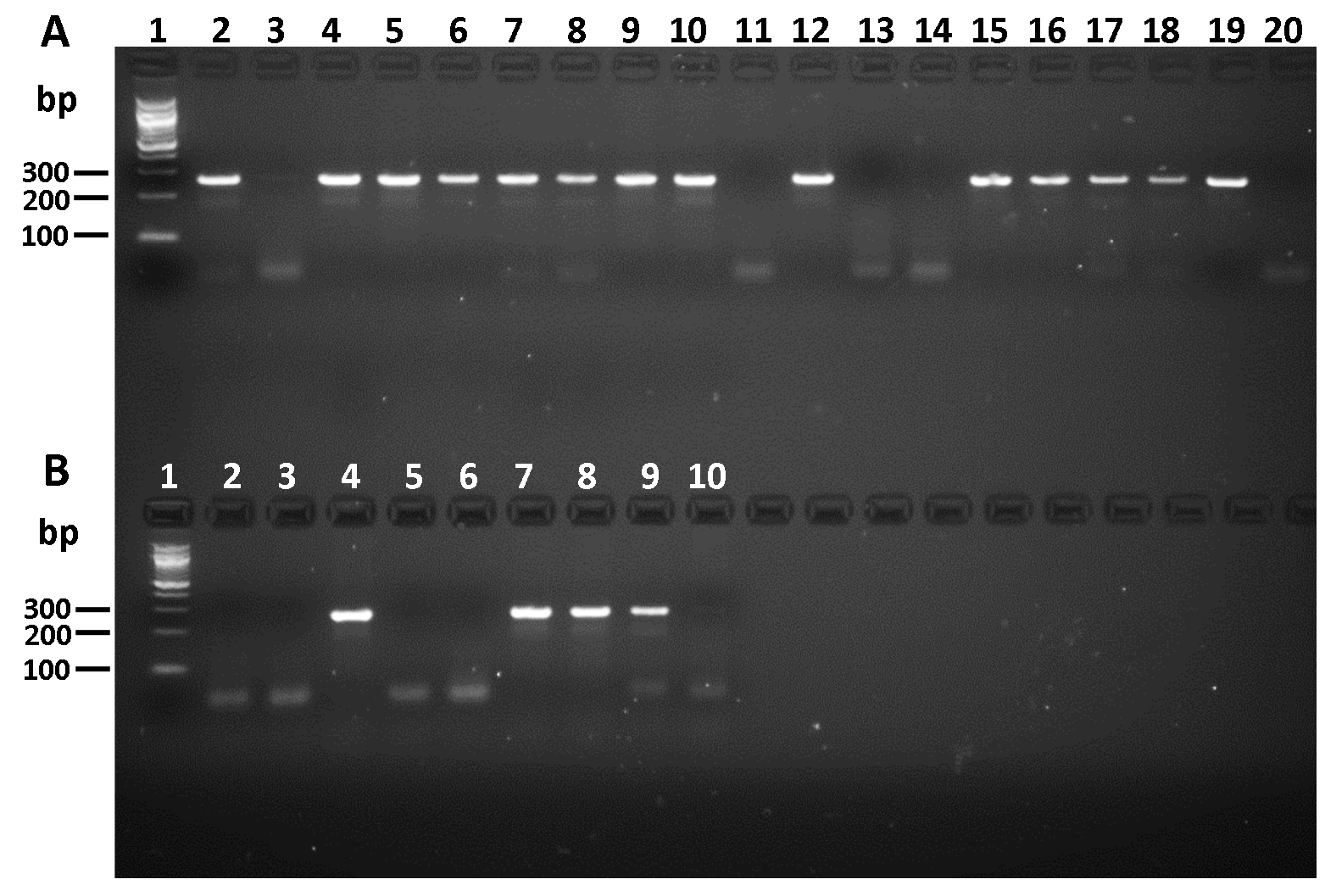
**

Figure 3.- *fimA* II genotype of *P. gingivalis* positives samples. Each lane corresponds to different patients. Lanes 20 and 10B are the negative controls. Lane 1A and 1B: 100-base pair DNA ladder marker
