# Supplementary material for: Periodontal pathogens and tetracycline resistance genes in subgingival biofilm of periodontally healthy and diseased Dominican adults
Source: Clin Oral Investig. 2015 Jun 30;20:349–56. doi: 10.1007/s00784-015-1516-2 (PMC4762914; doi:10.1007/s00784-015-1516-2)
Supplement: Supplementary file 4 — (DOCX 725 kb) [file 784_2015_1516_MOESM4_ESM.docx]

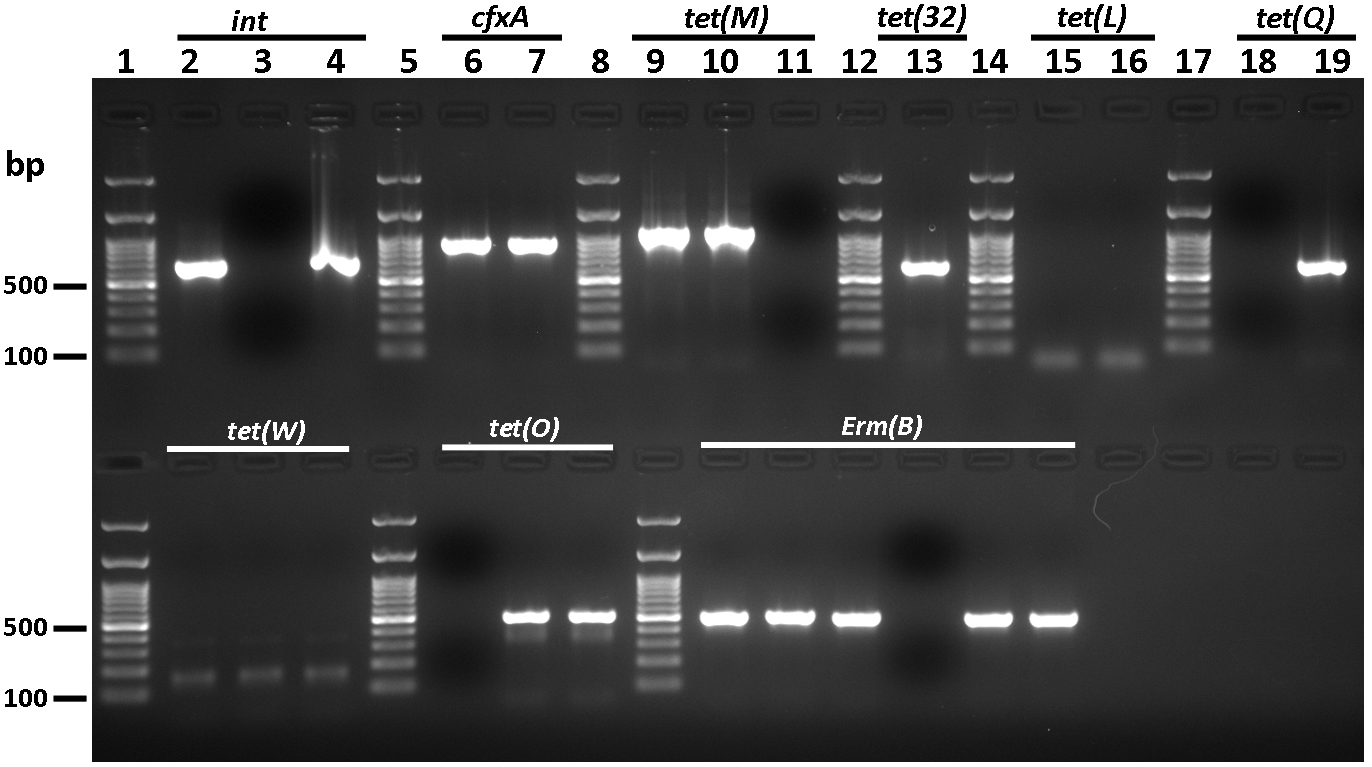


Figure 4.- Resistance gene detection using PCR in periodontal samples of Dominican Republic patients. Molecular weight standard corresponds to 100-base pair DNA ladder marker.
